# Implementation of Whole Genome Sequencing (WGS) for Identification and Characterization of Shiga Toxin-Producing *Escherichia coli* (STEC) in the United States

**DOI:** 10.3389/fmicb.2016.00766

**Published:** 2016-05-23

**Authors:** Rebecca L. Lindsey, Hannes Pouseele, Jessica C. Chen, Nancy A. Strockbine, Heather A. Carleton

**Affiliations:** ^1^Enteric Diseases Laboratory Branch, Centers for Disease Control and PreventionAtlanta, GA, USA; ^2^Applied Maths NVSint-Martens-Latem, Belgium; ^3^IHRC Inc.Atlanta, GA, USA

**Keywords:** *Escherichia coli*, whole genome sequence, STEC, next generation sequencing, *stx* subtyping, *Escherichia coli* serotypes

## Abstract

Shiga toxin-producing *Escherichia coli* (STEC) is an important foodborne pathogen capable of causing severe disease in humans. Rapid and accurate identification and characterization techniques are essential during outbreak investigations. Current methods for characterization of STEC are expensive and time-consuming. With the advent of rapid and cheap whole genome sequencing (WGS) benchtop sequencers, the potential exists to replace traditional workflows with WGS. The aim of this study was to validate tools to do reference identification and characterization from WGS for STEC in a single workflow within an easy to use commercially available software platform. Publically available serotype, virulence, and antimicrobial resistance databases were downloaded from the Center for Genomic Epidemiology (CGE) (www.genomicepidemiology.org) and integrated into a genotyping plug-in with *in silico* PCR tools to confirm some of the virulence genes detected from WGS data. Additionally, down sampling experiments on the WGS sequence data were performed to determine a threshold for sequence coverage needed to accurately predict serotype and virulence genes using the established workflow. The serotype database was tested on a total of 228 genomes and correctly predicted from WGS for 96.1% of O serogroups and 96.5% of H serogroups identified by conventional testing techniques. A total of 59 genomes were evaluated to determine the threshold of coverage to detect the different WGS targets, 40 were evaluated for serotype and virulence gene detection and 19 for the *stx* gene subtypes. For serotype, 95% of the O and 100% of the H serogroups were detected at > 40x and ≥ 30x coverage, respectively. For virulence targets and *stx* gene subtypes, nearly all genes were detected at > 40x, though some targets were 100% detectable from genomes with coverage ≥20x. The resistance detection tool was 97% concordant with phenotypic testing results. With isolates sequenced to > 40x coverage, the different databases accurately predicted serotype, virulence, and resistance from WGS data, providing a fast and cheaper alternative to conventional typing techniques.

## Introduction

Foodborne bacteria pose a major threat to public health. To prevent widespread infections due to these bacteria as well as detect outbreaks, rapid and accurate identification and subtyping of these bacteria is key. Shiga toxin-producing *Escherichia coli* (STEC) is an important foodborne pathogen estimated to cause over 265,100 illnesses each year in the United States (Scallan et al., 2011). STEC may present as a mild gastroenteritis, diarrhea, grossly bloody diarrhea and hemolytic uremic syndrome (HUS), and infection may be fatal. In the United States an estimated 96,500 O157 STEC and 168,690 non-O157 STEC infections occur each year and result in over 3600 hospitalizations and 30 deaths annually (Scallan et al., 2011).

STEC is a nationally reportable disease in the U.S. and clinical laboratory requirements for forwarding the STEC positive isolate or specimen to the public health laboratory vary by state. Once a STEC positive isolate or specimen arrives at the local or state public health laboratories it undergoes further characterization. These isolates are routinely subtyped using pulsed-field gel electrophoresis (PFGE) and submitted to the national surveillance network for foodborne bacteria, PulseNet, as well as characterized using conventional techniques for phenotype, serotype, and virulence. Workflows at public health laboratories for characterization for STEC can vary, current methods for characterization of STEC in the Enteric Diseases Laboratory Branch at the Centers for Disease Control and Prevention include panels of 22–49 phenotypic tests for identification, agglutination assays with 270 pooled and individual O- and H-specific antisera for serotyping (determination of 188 O and 53 H antigens), panels of five to 10 PCR assays for virulence profiling and broth microdilution assays for antimicrobial susceptibility testing. These methods require complex workflows, expensive reagents, labor-intensive quality control procedures, specialized training, and typically take 1–3 weeks to complete. Therefore a need exists to simplify workflows and reduce costs and time associated with subtyping and characterization of STEC, possibly through whole genome sequencing.

Whole genome sequencing (WGS) using benchtop instruments makes WGS possible in a public health lab setting. These machines are relatively easy to operate; the cost per isolate is low; and turnaround time for generating WGS data is within days rather than the 1–3 weeks required for current, conventional methods. Since the serotype, virulence and antimicrobial resistance profile may be predicted from the genome sequence, WGS may replace almost all reference characterization of STEC in the public health laboratory. Additionally, the sequence data also provide a level of strain discrimination and precision that is better than any subtyping method hitherto used for outbreak detection and investigation. Thus, almost all characterization of STEC in the public health laboratory can be replaced by WGS using one single efficient workflow. However, converting the WGS data into interpreted output that is useful for public health professionals is a real challenge.

To address this challenge, The Center for Genomic Epidemiology (CGE) (www.genomicepidemiology.org) has developed a suite of web-based tools for *in silico* analysis of bacterial whole genome sequence (Cosentino et al., 2013; Joensen et al., 2014). These tools include a serotype detection procedure (SerotypeFinder) and resistance and virulence prediction tool (ResFinder and VirulenceFinder) for analysis of *E. coli* and other bacterial WGS data (Zankari et al., 2013; Kleinheinz et al., 2014; Joensen et al., 2015). To characterize an isolate, WGS is uploaded to the website, and depending on the analysis requested, a report of the isolate's serotype, virulence, and resistance gene content is returned within several minutes to hours. Since many WGS analysis tools will accept data of any quality, it is important to understand the data quality requirements for the information being sought to interpret negative results correctly.

Although the CGE tools are useful for analysis in a setting where the isolate throughput is low and data analysis is centralized, for WGS analysis tools to be effective in a public health laboratory setting that processes tens to hundreds of WGS isolate sets per week, all the tools need to be merged into a single platform that performs WGS quality assessment and can also be used with a database that includes sufficient patient and sample information about the isolates to be able to interpret them in the proper epidemiological context, e.g., the outbreak setting. Moreover, the platform needs to be simple and user-friendly so that it may be used by public health professionals with limited bioinformatics skills. While numerous commercial and public domain software are available to analyze WGS data, very few combine databasing, WGS and other analytical capabilities, which are highly desirable in national and international laboratory surveillance networks. One exception is BioNumerics v7.5® (Applied Maths, Austin, TX), which is a commercial, customizable WGS quality assessment, analysis, and database software package that may be used for all these purposes. Thus the serotype, virulence, and resistance gene detection tools from CGE and *in silico* PCR tools to confirm results from virulence prediction tools can be integrated into a single push button tool in BioNumerics v7.5®, the genotyping plug-in. This plug-in can be used in the same database that contains the sequence data for subtyping for surveillance purposes and only requires a *de novo* assembled genome. Therefore, within the one software platform, reference characterization and WGS analysis for outbreak detection can be performed rapidly and requires little bioinformatics training for the user.

In this study, we performed validation of a genotyping plug-in within BioNumerics for identification of O and H genes using a diversity set of nearly 200 genomes. We further demonstrated the utility of the detection tools within the genotyping plug-in on isolates that were sequenced in-house and tested by traditional methods for serotype, virulence gene content, and antimicrobial susceptibility. In addition, we down-sampled the sequence reads of the later set to determine the minimum genome coverage needed for the program to detect the intended target genes and present *in silico* PCR tools to confirm selected results of the virulence detection tool.

## Materials and methods

### Whole genome sequencing and analysis

Validation of the genotyping plug-in within BioNumerics version 7.5 was performed using whole genome sequences of nearly 300 in-house sequenced and publically available STEC genomes for which conventional serotyping results were also available. The sequence data were generated on PacBio, Roche 454, and Illumina sequencing platforms (Table S1). An additional 17 genomes of *Shigella* and corresponding antimicrobial susceptibility testing data were included to evaluate the resistance finder tool. Moreover, a set of 106 isolates with traditional serotype and virulence PCR results performed in house were sequenced on the Illumina MiSeq or HiSeq sequencer platforms and selected for the down sampling experiment (Table S1). For in-house sequenced isolates, DNA was extracted using the Qiagen Blood and Tissue kit, libraries were prepared using NexteraXT (MiSeq) or NEB Next (HiSeq), and sequenced on the MiSeq or HiSeq using 2 × 250 bp chemistry. Sequence quality was evaluated on a per genome basis using BioNumerics version 7.5®. All genomes passed the basic quality metrics for raw sequence data from Illumina sequencers of average Q-score >30 in both reads and at least 40X average coverage with expected genome size for *E. coli* of 5 Mb. Read files of in house generated sequence data were uploaded to NCBI SRA using BioNumerics v.7.5 NCBI uploader (see Table S1). Genomes were processed through the BioNumerics Calculation Engine for *de novo* assemblies using the wgMLST client plug-in. The assembly was done using SPAdes version 3.5.0 integrated into the wgMLST plug-in and basic assembly metrics were calculated and used for quality assessment.

### Analysis using genotyping plug-in

Assembled sequence data was analyzed using the genotyping plug-in. The genotyping plug-in contains databases for serotype, virulence and resistance prediction (consisting of annotated allelic variants for genes encoding serotype, virulence factors and antimicrobial resistance), and for plasmid and prophage detection obtained from the Center for Genomic Epidemiology (DTU, Lyngby, Denmark) (https://cge.cbs.dtu.dk/services/data.php). The genotyping plug-in also contains an *in silico* PCR tool for the detection of Shiga toxin gene subtypes and virulence genes using previously published primers (Paton and Paton, 1998; Scheutz et al., 2012). The various “finder” tools use a blast-based approach to detect the genes of interest in the *de novo* assembled genome, and subsequently identifies them against the appropriate reference database. Detection parameters were set to 90% sequence identity and 60% sequence coverage. As a quality metric and a guard against blindly extrapolating the serotype, virulence or resistance prediction, for each similarity-based association, a discrimination score is calculated, indicating how good the closest known allele in the respective database fits the sample data with respect to the runner-up allele. The *in silico* PCR tools, mimicking the wet lab PCR process, excise a particular part of the genome, defined by forward and reverse primer pairs. In detecting a primer, at most 1 mismatch was allowed.

### Downsampling analysis

A set of 59 genomes were downsampled. A set of 40 genomes were used to validate the serotype and virulence gene finder, and the remaining set of 19 were used to validate the *stx* subtyper. The genomes were downsampled to 40x, 30x, 20x, and 10x coverage using the Computational Genomics Pipeline (CG-Pipeline; https://github.com/lskatz/CG-Pipeline; Katz et al., 2011). Downsampled genomes were assembled and analyzed using the genotyping plug-in BioNumerics v.7.5 with the settings outlined above to determine limit of detection for WGS-based identification tools.

### Conventional testing procedures

Conventional testing of isolates was completed in the *Escherichia Shigella* reference laboratory at the Centers for Disease Control and Prevention, USA. Serotyping was performed with O- and H-specific antisera from the Statens Serum Institut (Copenhagen, Denmark) by standard methods in a microtiter format (Ewing, 1986). For the virulence genes real-time or conventional PCR for the presence of Shiga toxin 1 and 2 (*stx*_1_, *stx*_2_), *stx* subtyping (*stx*_1a_, *stx*_1c_, *stx*_1d_, *stx*_2a,_ *stx*_2c,_ *stx*_2d,_ *stx*_2e_, *stx*_2f,_ and *stx*_2g_), intimin (*eae*) and hemolysin (*ehxA*) genes was performed (Paton and Paton, 1998; Scheutz et al., 2012). Broth microdilution assays to determine antimicrobial susceptibility was done by the National Antimicrobial Resistance Monitoring (NARMS) laboratory using previously published techniques (CDC, 2013). Resistance data were interpreted using Clinical Laboratory Standards Institute criteria (Clinical Laboratory Standards Institute, 2012).

## Results

### Validation of the serotype detection tool in bionumerics

The serotype detection tool within BioNumerics was validated on a total of 188 isolates for which WGS data and conventional serotype information was available. These publically available genomes were sequenced by either PacBio, Illumina, or 454 technology (see Table S1). The genomes represent 30 O serogroups and 26 H serogroups for a total of 76 serotypes (Table 1). Several representatives of the top 20 serotypes as well as a representation of a diverse collection of serotypes were selected for this set of genomes.

**Table 1:** **Validation of serotype detection tool within the genotyping plug-in on a set of 188 isolates**.

**Antigen**	**Number of strains phenotypically determined or reported in the literature with antigen**	**Number of strains with antigen determined by WGS in agreement with phenotypically determined antigen**
O1	3	2^b^
O2	2	2
O6	10	10
O7	4	4
O8	3	3
O9	3	3
O15	2	2
O16	7	7
O18	3	3
O25	3	3
O26	12	11^a^
O45	3	3
O55	20	20
O78	2	2
O83	2	2
O91	6	5^a^
O103	4	4
O104	4	4
O111	23	23
O118	1	0^b^
O119	1	1
O121	2	2
O127	1	1
O128	16	15^a^
O145	8	7^b^
O146	2	2
O149	2	1^a^
O157	36	35
O165	1	1
O174	3	3
H1	8	8
H2	18	18
H4	8	8
H6	13	13
H7	40	40
H8	5	4^b^
H9	2	2
H11	13	11^a^
H12	4	3^a^
H14	1	1
H16	2	2
H17	1	1
H19	6	6
H20	2	2
H21	16	14^a^
H25	4	4
H28	4	4
H31	1	1
H34	1	1
H37	1	1
H39	1	1
H43	4	4
H45	5	5
H47	1	0^a^
H48	7	7
H49	3	3

*Number of isolates given for positive by conventional and WGS serotype tests (isolate details listed in Table S1). 17 isolates were non-motile by traditional testing and not counted in the H WGS test results*.

a *Descrepant conventional and WGS serotyping results are noted by isolate in Table S1*.

b *Antigen not predicted from WGS data*.

A total of 29 O and 25 H serogroups were identified from the WGS data of the 30 O and 26 H serogroups detected by conventional methods, one O118 serogroup isolate was not detected by WGS data, and an H47 isolate was typed as an H7 by WGS. Comparisons to the traditional O serogroup results with the predictions from the WGS data showed that 96.3% (181/188) of the O serogroups were accurately predicted from the WGS data. For the H serogroup, 95.9% (164/171) of the H antigens were accurately predicted from the WGS data, H antigen detection for non-motile isolates was not counted since such isolates are non-typable by the phenotypic methods (Table 1). There were only 4 isolates that had a different O serogroup predicted and 6 isolates that had a different H serogroup predicted compared to traditional typing results (see Table S1). These were not sequenced by us but were downloaded from NCBI and it is possible that the data on NCBI may contain errors. However, since we do not have access to these isolates we cannot confirm the WGS results and phenotype. Overall, the serotyper tool predicted the serotype correctly in 94.2% (161/171) of the tested genomes.

### Robustness of serotype and virulence gene prediction in WGS datasets

To determine the sensitivity of the serotype and virulence gene predictions by WGS, a set of 40 isolates was selected that had all been sequenced by Illumina MiSeq or HiSeq and serotyped and virulence gene characterized using PCR methods. These genomes ranged in coverage from 40x to 267x coverage. Using the serotype detection tool in the genotyping plug-in, all but one of the O serogroups were predicted (95%) (38/40 isolates), both isolates belonging to the O153 serogroup were not predicted (Table 2). For those isolates where no O serogroup was predicted, the genomes ranged from 119x to 153x coverage, suggesting that sequence coverage was not a factor in being able to predict this particular serogroup. All of the H serotypes were predicted correctly when considering motile isolates, i.e. isolates that could be phenotypically verified by agglutination. The sequencing reads per isolate were then randomly down sampled to 40, 30, 20, and 10 times coverage and then analyzed in BioNumerics. These genomes were assembled *de novo* and the serotype and virulence genes predicted. In the down-sampled datasets, at 40x coverage 77.5% of O and 100% of H serogroups were correctly identified. For the remaining 30x, 20x, and 10x coverage levels, O serogroups were predicted correctly in 77.5, 52.5, and 17.5% of isolates and H serogroups were predicted in 100, 95, and 70% of isolates, respectively. The best prediction of O and H serogroup was from genomes at greater than 40x coverage.

**Table 2:** **Limit of detection for O and H antigens in a downsampled WGS data set from 40 strains**.

**Antigen^b^**	**Number of strains with phenotypically determined antigen**	**Number of strains by WGS having the phenotypically determined antigen at different sequence coverage levels**				
		**Original sequence coverage^a^**	**40x coverage**	**30x coverage**	**20x coverage**	**10x coverage**
O5	2	2	2	2	2	1
O26	2	2	2	2	1	0
O45	2	2	2	2	1	1
O69	2	2	2	2	1	0
O71	2	2	2	2	2	2
O76	2	2	2	2	2	1
O80	2	2	2	2	1	0
O91	2	2	2	2	0	0
O103	2	2	2	2	2	0
O104	2	2	2	2	0	0
O111	2	2	2	2	2	0
O113	2	2	2	2	1	0
O118	2	2	1	2	1	0
O121	2	2	2	2	2	2
O145	2	2	0	0	0	0
O146	2	2	1	1	1	0
O153	2	0	0	0	0	0
O157	2	2	2	2	2	0
O165	2	2	0	0	0	0
O174	2	2	2	0	0	0
H2	7	7	7	7	7	6
H4	3	3	3	3	3	2
H7	3	3	3	3	3	2
H8	2	2	2	2	2	2
H11	5	5	5	5	5	4
H14	1	1	1	1	1	1
H16	2	2	2	2	2	2
H19	4	4	4	4	4	2
H21	3	3	3	3	3	2
H25	1	2	2	2	2	1
H28	3	3	3	3	2	2

*Strain identifiers listed in Table S1*.

a *Original coverage ranged from 40 to 267x*.

b *Six H serogroups were called from the WGS data that were typed as non-motile by conventional methods and not included here*.

For the original sequence and 40x, 30x, and 20x down sampled genomes, there was 100% concordance between the virulence detection tool and *in silico* PCR and conventional real-time PCR assay for Shiga toxin 1 and 2 (*stx*_1_*, stx*_2_), intimin (*eae*), and hemolysin (*ehxA*) genes when a call was made (see Table 3). At 10x coverage, few virulence genes were identified from the WGS. The virulence detection databases did not identify *stx*_2_ in a STEC O76:H19 isolate which was detected by *in silico* PCR. Additionally, the *in silico* PCR did not identify *stx*_2_ in two isolates that were identified as *stx*_2_ positive by the virulence detection tool databases. By using both the virulence detection databases and *in silico* PCR, all *stx*_2_ positive isolates identified by conventional typing methods were also identified from the WGS at 20x coverage or higher. For the other virulence gene targets, *stx*_1_ was detected in all 21 of the isolates positive by conventional testing at ≥ 20x coverage, *eae* in all 26 at ≥ 30 coverage, and *ehxA* gene detection from WGS data was 100% concordant in the assemblies from the original sequence read set.

**Table 3:** **Limit of detection of virulence genes in a down sampled WGS data set from 40 STEC and one hybrid STEC/EAEC O104:H4 by both a blast and *in silico* PCR approach**.

**Trait**	**Number of strains with trait by real-time PCR**	**Number strains determined from WGS data with indicated trait at different sequence coverage levels**									
		**Original sequence coverage**^**a**^		**40x coverage**		**30x coverage**		**20x coverage**		**10x coverage**	
		**Blast**	***In silico* PCR**	**Blast**	***In silico* PCR**	**Blast**	***In silico* PCR**	**Blast**	***In silico* PCR**	**Blast**	***In silico* PCR**
*stx1*	27	27	27	27	27	27	27	27	27	25	26
*stx2*	21	20^b^	19^c^	20	19	20	20	20	20	20	18
*eae*	26	26	26	26	24	26	26	24	20	8	9
*ehxA*	29	29	29	26	26	25	25	17	20	8	11

*Strain identifiers listed in Table S1*.

a *Original coverage was 40x to 267x*.

b *For the original sequence files, stx2 was missed in an E. coli O76:H19 using the genotyping plug-in that was detected by in silico PCR*.

c *The in silico PCR did not detect stx2 in 2 isolates though it was detected by the genotyping plug-in*.

### Prediction of *stx* gene subtype using virulence gene database and *in silico* PCR

A total of 19 isolates were examined that had complete conventional *stx* gene subtype results and WGS results. These isolates represented the a, c, and d subtypes of *stx*_1_ and the a, b, c, d, e, f, and g subtypes of *stx*_2_. All the *stx* subtypes except *stx*_2c_ were detected using the *in silico* PCR tool or the virulence detection database at original coverage levels and down to 10x coverage (see Table 4). For *stx*_2c_, only one of the two isolates identified as positive by conventional laboratory testing was detected as positive from the WGS using *in silico* PCR. One isolate was positive for *stx*_1a_ using blast against the virulence gene database but was negative by both conventional testing using PCR and the *in silico* approach. Looking further into this discrepancy, using the blast based approach the gene was only a 82.3% length match compared to the reference allele and may indicate the gene was truncated so that the reverse primer used in the traditional or *in silico* PCR assay would not hybridize. Overall, *stx* gene subtype was correctly predicted in 89.5% of isolates at ≥ 10x coverage.

**Table 4:** **Limit of detection of *stx* gene subtype in a downsampled WGS data set for 19 strains by both a blast and *in silico* PCR approach**.

**Trait**	**Number of strains with trait by real-time PCR**	**Number strains determined from WGS data with indicated trait at different sequence coverage levels**									
		**Original sequence coverage**		**40x coverage**		**30x coverage**		**20x coverage**		**10x coverage**	
		**Blast**	***In silico***	**Blast**	***In silico***	**Blast**	***In silico***	**Blast**	***In silico***	**Blast**	***In silico***
			**PCR**		**PCR**		**PCR**		**PCR**		**PCR**
*stx_1*a*_*	2	3	2	2	2	2	2	2	2	2	2
*stx_1*c*_*	1	1	1	1	1	1	1	1	1	1	0
*stx_1*d*_*	2	2	2	2	2	2	2	2	2	2	1
*stx_2*a*_*	2	2	2	2	2	2	2	1	2	2	1
*stx_2*b*_*	3	2	3	2	3	2	3	3	3	3	2
*stx_2*c*_*	2	0	1	0	1	0	1	0	0	0	0
*stx_2*d*_*	3	3	3	3	3	3	3	3	2	3	2
*stx_2*e*_*	1	1	1	1	1	1	1	1	1	1	1
*stx*_2*f*_	7	7	7	7	7	7	7	7	7	7	6
*stx_2*g*_*	1	1	1	1	1	1	1	1	1	1	1

*see Table S1 for isolate identification*.

### Validation of the resistance finder tool in bionumerics

The resistance finder tool in BioNumerics was evaluated against a set of 46 isolates where WGS and traditional antimicrobial susceptibility results were available. Several of the isolates tested, a total of 28 out of the 46, were pan-susceptible by both antimicrobial susceptibility testing and did not contain resistance genes by WGS (see Table 5). Of the remaining STEC and *Shigella* that were found to be resistant by traditional antimicrobial susceptibility testing, the concordance for detecting genetic antimicrobial resistance determinants for ampicillin, azithromycin, chloramphenicol, sulfisoxazole, streptomycin, and trimethoprim/sulfamethoxazole was 100%. One isolate was found to contain tetracycline resistance genes that did not test as resistant by conventional testing. No genetic resistance determinants were detected for isolates resistant to nalidixic acid and ciprofloxacin using the ResFinder database. Through further genetic analysis, it was determined that these isolates were resistant via chromosomal mutations in the *gyrA* gene alone or in combination with mutations in the *parC* gene. These results are not unexpected as gene detection schemes can identify non-functional genes and do not detect mutational events. Taking these issues into account, there was 99.7% concordance between phenotypic susceptibility and antimicrobial resistance detection by WGS.

**Table 5:** **Comparison of phenotypic antimicrobial susceptibility testing results with resistance determinants identified from WGS data in 46 strains**.

**Antimicrobial**	**Phenotypic laboratory testing**	**Resistance determinant detection from WGS data**
Ampicillin	12	12
Azithromycin	13	13
Chloramphenicol	1	1
Streptomycin	18	18
Sulfisoxazole	18	18
Nalidixic acid	9	0
Ciprofloxacin	6	0
Trimethoprim/Sulfamethoxazole	18	18
Tetracycline	17	18
No Resistance detected	28	28

*Values indicate the number of strains identified with resistance to the indicated antimicrobial*.

## Discussion

In this study, we demonstrated the utility and accuracy of a single software platform for combining workflows for quality assessment and reference characterization of STEC through WGS data. A single software program that can be used by non-bioinformaticians is a requirement for public health professionals to be able to infer phenotypic results from WGS data. Using publically available databases and *in silico* PCR tools developed as part of this study, we identified the same information (serotype, virulence genes, and resistance determinants) from the WGS data for 94.7% *E. coli* and *Shigella* isolates as was identified previously by conventional methods. Additionally, the limit of detection for these determinants was established through down sampling experiments allowing for better interpretation of negative results and understanding of sequence data quality needed for reference characterization from WGS.

Although, other recent studies have already shown the utility of reference characterization directly from sequence data generated from benchtop sequencers (Joensen et al., 2014, 2015; DebRoy et al., 2016), we present the combined quality assessment, serotyping, virulence profile, and resistance profile in one simple, high-throughput, and user-friendly analytical WGS workflow. These previous studies extensively validated their findings against those obtained by conventional methods, yet limited testing was done to identify the limit of detection for these tools and how best to interpret a negative result. Often in these studies, sequences were selected because they were of high quality and had high coverage, typically over 50x. While high sequence quality and coverage may be possible during routine testing periods, it is often difficult to achieve during outbreak response or when trying to reduce testing costs. When there is a need to increase isolate multiplexing per sequencing run to increase throughput and reduce costs, sequence coverage per isolate decreases. For this reason we attempted to determine the limits for coverage to help determine the maximum number of isolates that could be sequenced at the same time. In our study, sequence coverage of ≥30 was enough to predict 100% of the H serogroups, for the O serogroups 93% of the serogroups were correctly predicted at a sequence coverage > 40x. One O antigen, O153 (2 isolates), was not detected in genomes sequenced to over 100x coverage. Since other groups have shown that O153 genes *wzx* and *wzy* are 100% identical to the O178 genes, even though surprisingly these two serogroups are not cross-reactive using phenotypic testing, the current similarity-based WGS detection methods may not be able to distinguish these closely-related serogroups (Joensen et al., 2014, 2015; DebRoy et al., 2016).

Virulence gene detection performed more robustly than O and H antigen gene detection. The majority of virulence genes were detected at ≥20x coverage in the WGS data using the genotyping plug-in. Since both serotype and virulence gene information is needed for STEC surveillance, to be able to consistently identify serotype from WGS in all isolates >40x coverage is recommended. Preliminary data (not presented) indicates that this coverage will also suffice for subtyping for outbreak investigations using whole genome multilocus sequence typing analysis.

To improve confidence in negative WGS results, additional *in-silico* PCR tools were developed to double check negative results from whole genome sequence data. This provided further confidence in virulence typing results. By using both WGS typing tools, all virulence genes were detected, by relying on either tool alone, blast algorithm or *in silico* PCR, important virulence genes would have been missed. Being able to accurately identify virulence genes and *stx* gene subtypes is important because certain virulence gene combinations are associated with higher risks for adverse events, e.g., HUS (Scheutz et al., 2012). Other groups have also shown the robustness of determining *stx* gene subtypes from WGS from both O157 and non-O157 serogroups (Ashton et al., 2015; Chattaway et al., 2016).

For identification of resistance determinants, the ResFinder database produced highly concordant results with traditional phenotypic testing. Isolates possessing quinolone resistance mechanisms that were not identified by the ResFinder database, underscore the limitations of a gene-based detection approach. Supplementary *in silico* PCRs using conventional primer sets (Conrad et al., 1996; Bhattacharya et al., 2013) and subsequent sequence analysis or reference-mapping tools can be used in conjunction with the resistance finder tool, in order to detect mutational events conferring antimicrobial resistance. A recent study examining multi-drug resistant *E. coli* in the United States accurately predicted drug resistance with high specificity and sensitivity using a WGS approach that employed gene-based detection in conjunction with mutational analysis of the quinolone resistance-determining regions of the chromosome (Tyson et al., 2015). The present study confirms this high concordance between genetic and phenotypic testing for antimicrobial resistance, and also reveals the ability of this WGS-based approach to distinguish resistant and susceptible isolates for most drug classes.

From this study, it has been shown that quality assessment, serotyping, virulence and resistance profiling can be performed in one simple workflow. Additionally, the information that is extracted from WGS has more details than provided by conventional methods, e.g., by conventional methods we routinely detect only 5 virulence targets and 9 antimicrobial susceptibilities whereas over 100 virulence and resistance determinant genes are detected by WGS. Extracting this information from the whole genome sequence rather than using traditional identification techniques is highly cost-efficient: it is possible to save up to 180 US dollars on reagents alone per characterized isolate (assuming $123 for WGS and $304 for traditional typing workflow per isolate), which makes WGS both more rapid and less expensive for typing STEC. Additionally other groups (Joensen et al., 2014) have shown that the turnaround time for WGS is faster compared to conventional reference identification and subtyping workflows. We have also seen that the turnaround time from receipt of isolate in lab to WGS result can be 3–4 days while conventional methods take 1–3 weeks. While there is a high initial overhead cost of the sequencing instrument, WGS has the potential to streamline laboratory work into a unified workflow, reducing the need for multiple specialized personnel and instruments for various genotypic and phenotypic testing. Furthermore, by understanding the limit of detection by WGS for these different targets, there is more confidence that a negative result is an accurate prediction, though further work needs to be done to fine tune the serotype and resistance finder databases.

## Future work

For future work on the genotyping plug-in within BioNumerics, we plan to integrate more gene identification techniques from the whole genome sequence, such as reference mapping, to improve the ability to detect serogroups and resistance determinants. Lastly, although this validation was done for reference characterization and a minimum coverage requirement was identified, similar tests need to be performed in terms of WGS analysis for outbreak detection. Currently we are validating a whole genome multilocus sequence typing (wgMLST) database for STEC.

## Author contributions

RL, HC designed this work, RL, HC, JC, and HP contributed to the analysis and interpretation of the work. RL and HC drafted the manuscript and RL, HC, JC, HP, and NS reviewed the manuscript for intellectual content.

## Funding

This work was made possible through support from the Advanced Molecular Detection (AMD) initiative at the Centers for Disease Control and Prevention.

### Conflict of interest statement

The authors declare that the research was conducted in the absence of any commercial or financial relationships that could be construed as a potential conflict of interest. HP is affiliated as an employee (chief operations officer) with the following organization: Applied Maths NV, Keistraat 120, B-9830 Sint-Martens-Latem, Belgium.
